# Efficacy and safety of baricitinib in patients with severe COVID-19: A systematic review and meta-analysis

**DOI:** 10.1097/MD.0000000000036313

**Published:** 2023-12-01

**Authors:** Wenxin Song, Shishen Sun, Yilong Feng, Liujun Liu, Tianqi Gao, Shaoxiang Xian, Jie Chen

**Affiliations:** a The First Clinical Medical College of Guangzhou University of Chinese Medicine, Guangzhou, China; b Guangzhou University of Chinese Medicine, Guangzhou, China; c The First Affiliated Hospital of Guangzhou University of Chinese Medicine, Guangzhou, China.

**Keywords:** baricitinib, efficacy, meta-analysis, safety, severe COVID-19

## Abstract

**Background:**

This study aimed to investigate the efficacy and safety of baricitinib in patients with severe coronavirus disease 2019 (COVID-19).

**Methods:**

Databases were searched for studies that compared the clinical efficacy and adverse effects of baricitinib with standard therapy for the treatment of severe COVID-19 and clearly reported relevant outcomes published until December 31, 2022. The corresponding data were extracted from these studies. A fixed-effects model was used to calculate the pooled estimates. The study protocol can be accessed at PROSPERO (CRD42023394173).

**Results:**

The baricitinib group had a significantly lower mortality rate and proportion of patients who received mechanical ventilation than the control group (OR = 0.61, 0.57; *P* = .008, 0.02; 95% CI 0.42–0.88; 0.35–0.92; I^2^ = 71% and 86%, respectively). The length of hospital stay and rates of severe adverse events were not significantly different between the 2 groups.

**Conclusion:**

Baricitinib reduces mortality and mechanical ventilation requirements in patients with severe COVID-19. Therefore, we developed a comprehensive understanding of the role of baricitinib in patients with severe COVID-19.

## 1. Introduction

Coronavirus Disease 2019 (COVID-19) is a public health emergency of international concern declared by the World Health Organization.^[1]^ To date, there have been 753 million cases of COVID-19 and 6.8 million deaths globally.^[2]^ COVID-19 imposes a heavy economic burden and increases disability and morbidity rates.

Dysregulation of the immune system in COVID-19 patients has been linked to poor prognosis.^[3]^ Elevated levels of inflammatory markers, including C-reactive protein and interleukins (e.g., IL-1 and IL-6) in the later stages of viral infection indicate the immune origin of worsening respiratory symptoms.^[4]^ Therefore, in addition to antivirals, immunomodulators are considered adjunctive therapies for the management of severe COVID-19 immune overactivation. Adrenocortical hormones and various immunomodulators play a role in the management of COVID-19, including Janus-kinase/signal transducers and activators of transcription (JAK-STAT) inhibitors, IL-6 inhibitors, and IL-1 receptor blockers.^[5]^

Baricitinib, a drug approved by the U.S. Food and Drug Administration for the treatment of active rheumatoid arthritis, was recently identified as a new hope for the treatment of severe pneumonia from COVID-19, with the Food and Drug Administration Emergency Use Authorization (EUA) to use its 4 mg dose in COVID-19 on November 19, 2020. Baricitinib, an inhibitor of Janus kinases JAK-1 and JAK-2, plays a dual role in inhibiting the excessive inflammatory response of COVID-19 pneumonia, including inhibiting the release of pro-inflammatory mediators and endocytosis of the virus.^[6]^ Cell-mediated signaling pathways between JAKs and STATs are key to cytokine release. Baricitinib blocks this pathway by inhibiting JAK-1 and JAK-2, thereby downregulating the inflammatory cytokine storm in COVID-19, and may have additional antiviral activity.^[6]^

Several small cohort observational studies of patients hospitalized with COVID-19, including older patients, have associated clinical improvement with baricitinib treatment.^[7–9]^ In an exploratory, randomized, placebo-controlled trial of severely hospitalized COVID-19 patients requiring invasive mechanical ventilation or extracorporeal membrane oxygenation, baricitinib therapy significantly reduced 28-day all-cause mortality compared to placebo.^[10]^

However, the clinical experience with baricitinib in patients with severe COVID-19 remains limited. To date, no head-to-head trials have been conducted to assess the best anti-cytokine option for patients with severe disease. Additionally, the reported benefits of baricitinib treatment in patients with severe COVID-19 have been inconsistent between studies, making it difficult to conclusively estimate treatment efficacy. Therefore, we conducted a meta-analysis to compare the efficacy and safety of baricitinib treatment with the standard of care (SOC) in patients with severe COVID-19. This information can help clinicians in making clinical decisions when managing severe COVID-19 admissions.

## 2. Methods

### 2.1. Search strategy

We conducted this meta-analysis according to the Cochrane Collaboration and Preferred Reporting Items for Systematic Reviews and Meta-Analyses statements.^[11]^ MEDLINE, EMBASE, and Cochrane Central were searched up to December 31, 2022, using the following search terms: “baricitinib,”

“Janus kinase inhibitor,” “JAK inhibitor,” “severe,” “SARS-CoV-2,” “coronavirus,” “nCoV,” ‘pneumonia, ‘ “respiratory failure,” ‘corona- virus,‘ “2019 nCoV” and “COVID-19.” All literature was imported into EndNote version 20 to identify and remove duplicate results. We limited the results to human studies and the language to English.

### 2.2. Inclusion and exclusion criteria

We includes studies comparing the clinical efficacy and adverse effects of baricitinib with standard therapy in the treatment of COVID-19 that clearly report on at least one relevant outcome, including all-cause mortality, days of treatment with mechanical ventilation (MV), length of hospital stay, and adverse event rate. Studies were excluded if: they were case reports; they were single-arm studies; they did not report the efficacy of baricitinib in the treatment of COVID-19; they did not compare baricitinib with placebo or control groups; they were pharmacodynamic studies; or they were in vitro studies. To avoid bias, 2 authors (Tq G and Wx S) searched for and examined articles, respectively. If there was disagreement between the 2 authors, the third author helped to resolve the issue and make the final decision. Data were collected on the author, year of publication, study country, demographic characteristics of patients, protocol of the experimental and control groups, and duration of follow-up. The primary outcomes were the all-cause and adverse event rates. The secondary outcomes were the proportion of patients who received mechanical ventilation (MV) and days in hospital. This meta-analysis was registered in the PROSPERO database (CRD42023394173).^[12]^

### 2.3. Statistical analysis

We performed statistical analysis using Review Manager v.5.4 (Cochrane Collaboration). We evaluated statistical heterogeneity using the Cochran Q and I^2^ statistics. Significant heterogeneity was considered with *P* < .1 or I^2^ > 50%. Combined odds ratios (ORs) and 95% confidence intervals (CI) were calculated, and *P* < .05 was considered statistically significant. Studies may have reported either the medians or means of hospitalization duration and recovery time. To standardize this, the values were converted to mathematically implied means based on an exponential distribution, a standard assumption for time-to-event data. Finally, the GRADE approach (Grading of Recommendations Assessment, Development and. Evaluation) was used to assess the quality of generated evidence for the outcomes for which pooled analyses were performed.^[13]^

## 3. Results

### 3.1. Characteristics of the studies

**Figure 1.**
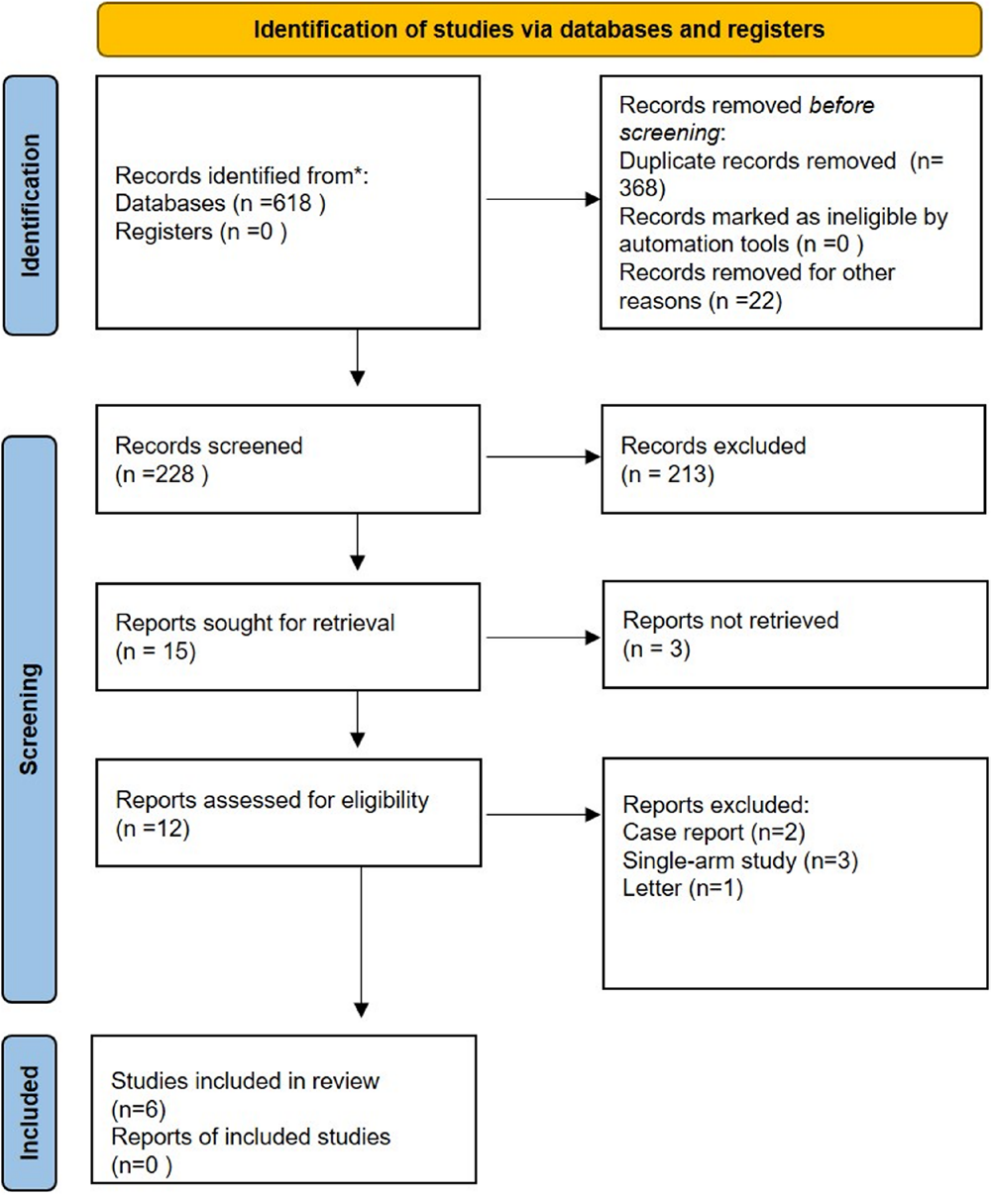
PRISMA flow chart outlining literature search. PRISMA = Preferred Reporting Items for Systematic Reviews and Meta-Analyses.

Figure 1 shows the Preferred Reporting Items for Systematic Reviews and Meta-Analyses flowchart that summarizes the search strategy. The search strategy initially produced 618 references, 228 of which were assessed after eliminating duplicate articles. Finally, after excluding 213 articles according to the title and abstract of the articles, we selected 12 articles for full-text review. According to the exclusion criteria, 6 articles were excluded after a full-text review. Finally, 6 studies^[10,14–18]^ were selected for inclusion in this meta-analysis. Overall, the meta-analysis included 920 patients, of whom 494 received baricitinib and 426 received standard care. All 6 included studies were single-center studies. Among those studies, one was a double-blind, placebo-controlled randomized controlled trial,^[10]^ 3 were prospective observational cohort studies,^[15,17,18]^ and 2 were retrospective studies.^[14,16]^ Detailed information included in this meta-analysis is shown in Table 1.

**Table 1:** Characteristics of included studies.

Author, yr	Trial countries	Treatment time (d)	Sample size		Age (yr)*		Number of patients (female)		Inclusion criteria	Exclusion criteria	Regimen of control group	Regimen of experiment-al group	Primary outcome†	Duration of follow-up
			Control group	Experimen-tal group	Control group	Experimen-tal group	Control group	Experimen-tal group						
E Wesley Ely, et al 2022^[14]^	Argentina, Brazil, Mexico, and the United states	14	50	51	58 ± 8	58 ± 4	20	26	Participants aged ≥ 18 yr of age with positive laboratory confirmed of SARS-CoV2 infection and use of IMV or ECMO at study entry and randomization and at least one elevated inflammatory marker greater than the upper limit of normal range based on the local laboratory result (C-reactive protein, D-dimer, lactate dehydrogenase, or ferritin).	Receiving high-dose corticosteroids for ≥ 14 consecutive days in the month before study entry; had major comorbidities such as asthma, chronic obstructive pulmonary disease, or adrenal insufficiency; had received convalescent plasma or intravenous immunoglobulin for COVID-19; or had suspected serious active bacterial, fungal, or other infection, or untreated tuberculosis infection.	Placebo for up to 14 d or until discharge from hospital and standard of care	Baricitinib 4mg/d up to 14 d or until discharge from hospital (whichever occurred first) and standard of care	①③④	28 d
Eduardo Perez-Alba, et al 2021^[19]^	Mexico	14	74	123	58.5 ± 16.5	60.7 ± 13.1	25	49	Participants aged > 18 yr with a positive RT-PCR for SARS-CoV-2 and at least one of the following: a respiratory rate of 30 or more breaths per minute, a blood oxygen saturation of 93% or less, a ratio of the partial pressure of arterial oxygen to the fraction of inspired oxygen (PaO2/FiO2) of <300 mm Hg, or pulmonary infiltrates in more than 50% of the lung fields.	Hospital stay <24 h; patients without oxygen requirement or patients that received any other therapies such as convalescent plasma, tocilizumab, remdesivir or methylprednisolone.	Dexamethasone 6 mg/d i.v. for 10 d and standard of care	Baricitinib 4 mg/d for 14 d and dexamethasone 6 mg/d i.v. for 10 d and standard of care	①②③④	NA
Jose Luis Rodriguez-Garcia, et al 2020^[18]^	Spain	3 + 10	50	62	64 (57,69)	63 (52, 72)	16	18	Participants admitted during the observation period with SARS-CoV-2 pneumonia and respiratory insufficiency (oxygen saturation as measured by pulse oximetry (SpO2) < 92% breathing room air)	Had major comorbidities (chronic heart failure, obstructive sleep apnea syndrome with continuous positive airway pressure, advanced chronic kidney disease, active malignancies); Admitted to ICU or died.	Received 3 consecutive days of pulse corticosteroid therapy (corticosteroids pulses) followed by prednisone at a starting dose of 30 mg/d.	Received corticosteroids for 3 d and then prednisone, combined with baricitinib for 5 to 10 d. Baricitinib: 4 mg the first day and then 2mg/d (n = 40) or 4/d (n = 22).	③④	1 mo after discharge
Mar Masiá, et al 2021^[20]^	Spain	14	95	95	72 (60, 80)	72 (62, 78)	39	35	Participants aged ≥ 18 yr with confirmed SARS-CoV-2 infection and abnormal findings on chest x-ray, and/or severity criteria, including oxygen saturation < 94% and CURB-65 ≥ 2.	Receipt of convalescent plasma or IV immunoglobulin for COVID-19; or suspected serious active infection or untreated tuberculosis infection	Tocilizumab plus dexamethasone plus remdesivir plus standard of care	Baricitinibs plus tocilizumab plus dexamethasone plus remdesivir plus standard of care	①②③④	12 mo
Md. Jahidul Hasan, et al 2021^[21]^	Bangladesh	14	116	122	59 (54,68)	63 (54.8, 69)	40	39	Participants with confirmed COVID-19 pneumonia lesions (bilateral ground-glass opacities) (> 50%) in the chest computerized tomography (CT) scan images at the time of admission and having at least 2 additional signs of severe: (I) dyspnea; (II)oxygen saturation in blood (SpO2) level ≤ 93% on room air; and (III) respiratory rate ≥ 30 breaths/min	Patient with pregnancy; any history of acute/chronic autoimmune disease or active/latent tuberculosis infection; history of hospital stay for > 3 d for any purpose with the last 3 mo; current evidence of bacterial or fungal coinfection	Baricitinib 4mg/d for 14 d plus dexamethasone 0.25 mg/kg/d i.v. plus remdesivir (200 mg loading followed by 100 mg once daily) plus standard of care	Baricitinib 8 mg/d for 14 d plus dexamethasone 0.25 mg/kg/d i.v. plus remdesivir (200 mg loading followed by 100 mg once daily) plus standard of care	①③④	NA
Takuya Tanimoto, et al 2022^[17]^	Japan	NA	41	41	69 (58, 78)	72 (57, 79)	14	10	Participants with respiratory failure associated with COVID-19	Death or transfer to another hospital within 3 d; history of advanced chronic kidney disease (estimated glomerular filtration rate [eGFR] < 15 mL/ min/1.73 m^2^); decompensated cirrhosis; or administration of biologics or other JAK inhibitors.	Drugs were given in various combinations at the discretion of the attending physician	Baricitinib plus other drugs were given in various combinations at the discretion of the attending physician	①②③④	NA

i.v. = intravenous, NA = not available.

* Expressed as mean ± standard deviation or median (interquartile range).

†① All-cause mortality within 60 d; ② proportion who received mechanical ventilation; ③ length of hospital stay, days; ④ severe adverse event rates (such as venous thromboembolism events, acute kidney failure, and severe allergies).

### 3.2. Risk of bias assessment

The revised Cochrane Risk Bias 2 tool was used to assess the risk of bias in one randomized controlled trial, which showed a moderate risk of bias.^[19]^ For the 5 observational cohort trials, the Newcastle–Ottawa scale was used to assess the risk of bias.^[21]^ Each study had a quality assessment score of 7, indicating a moderate risk of bias in all 5 studies.

### 3.3. Assessment of outcomes

**Figure 2.**
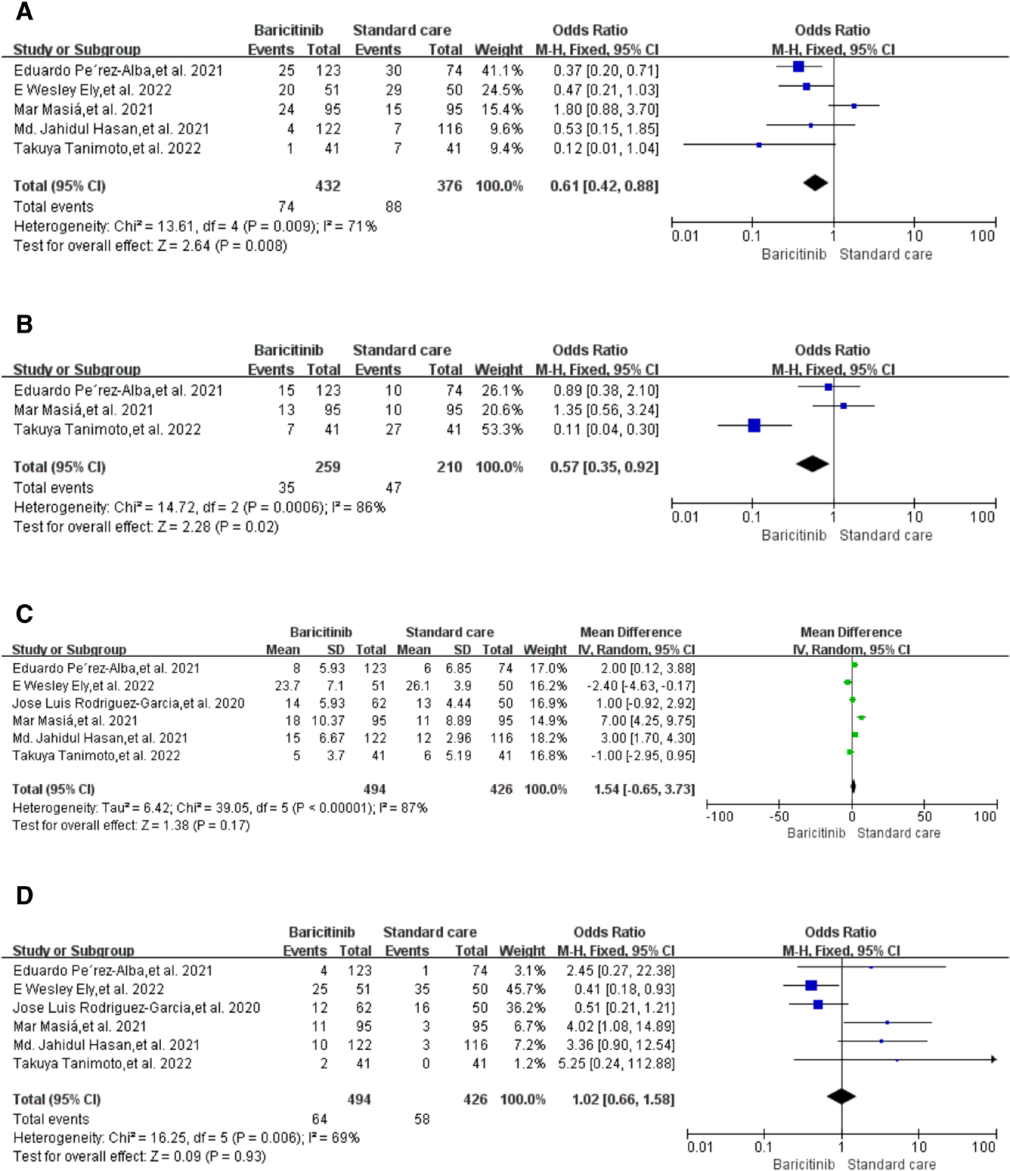
Forest plots for primary and secondary outcomes. (A) All-cause mortality within 60 d; (B) proportion who received mechanical ventilation; (C) length of hospital stay; and (D) severe adverse event rates.

Five studies^[10,14,16–18]^ reported all-cause mortality within 60 days. Pooled analysis showed that 432 participants in the baricitinib group had a significantly lower mortality rate than 376 participants in the control group (17.1% vs 23.4%; OR = 0.61, *P* = .008, 95% CI 0.42–0.88; I^2^ = 71%) (Fig. 2A).

Three studies^[14,16,17]^ reported the results of the proportion of patients who received mechanical ventilation. Combined analysis showed that 259 participants in the baricitinib group received less mechanical ventilation during hospitalization than 210 participants in the control group (13.5 vs 22.3%; OR = 0.57, *P* = .02, 95%CI 0.35–0.92; I^2^ = 86%) (Fig. 2B)

Six studies^[10,14–18]^ reported the length of hospital stay. Combined analysis showed that the length of hospital stay of 494 participants in the baricitinib group was similar to that of 426 participants in the control group, with no significant difference (OR = 1.54, *P* = .17, 95% CI -0.56–3.73; I^2^ = 87%) (Fig. 2C)

Six studies^[10,14–18]^ reported severe adverse events. Pooled analysis showed that the severe adverse event rates of 494 participants in the baricitinib group were similar to those of 426 participants in the control group, with non-significant differences (12.9% vs 13.6%; OR = 1.02, *P* = .93, 95% CI 0.66–1.58; I^2^ = 69%) (Fig. 2D). Quantitative analysis of publication bias and subgroup analysis was not performed because of the limited number of studies included in the meta-analysis.

The GRADE tables representing the quality of generated evidence for the outcomes for which pooled analyses were performed are provided in supplementary Table S1 to Table S4, http://links.lww.com/MD/K833, http://links.lww.com/MD/K834, http://links.lww.com/MD/K835, http://links.lww.com/MD/K836.

## 4. Discussion

To our knowledge, this meta-analysis is the first comprehensive analysis of clinical studies that focused on the efficacy and safety of baricitinib in patients with severe COVID-19 infection to date. The results of this study showed that the mortality rate and need for mechanical ventilation in patients with severe COVID-19 treated with baricitinib were significantly lower than those of standard treatment. Baricitinib treatment did not significantly alter the length of stay or incidence of serious adverse events in patients hospitalized with severe COVID-19 compared with standard treatment. However, according to another report, baricitinib or tocilizumab treatment resulted in significantly shorter hospital stays compared with patients receiving standard care.^[20]^ This may be related to the fact that the patients included in the study were all severely ill; the relatively small sample size highlights the importance of our meta-analysis.

IL-6 is a multifunctional cytokine secreted by neutrophils, monocytes, and macrophages and plays a key role in inflammatory responses. IL-6 promotes overactivation of the immune response.^[22]^ Elevated levels of these cytokines (especially IL-6) lead to impaired blood gas exchange in alveolar capillaries. This, in turn, leads to impaired oxygen diffusion followed by inflammation, which eventually leads to pulmonary fibrosis and multiple organ failure. Notably, elevated IL-6 levels have also been associated with hypercoagulability in patients with COVID-19.^[23]^ Baricitinib is an IL-6 receptor antibody that exhibits antiviral activity at a tolerable therapeutic dose range by inhibiting JAK1/JAK2 enzyme activity. It prevents the virus from entering cells by inhibiting INF-1, which is upregulated by the ACE-2 receptor.^[24]^ Thus, it can block the entry of suppressor cells through inhibition of clathrin-mediated endocytosis. As the JAK-STAT signaling pathway is central to the development of cytokine storms in severe COVID-19, baricitinib may help ameliorate its symptoms.^[25]^ The anti-cytokine and antiviral activities of baricitinib are the primary cause of the rapid reduction in clinical and radiological recovery, viral load, inflammatory markers, and IL-6 levels from COVID-19.

It is pertinent to mention that for the treatment of mild to moderate COVID-19, the efficacy of Paxlovid, which is FDA-approved and strongly recommended by the WHO, has recently been validated in numerous clinical trials.^[26]^ Treatment with Paxlovid in the first 5 days of SARS-CoV-2 infection is associated with a markedly reduced risk of progression to severe COVID-19 or mortality, regardless of the vaccination status for SARS-CoV-2.^[27]^ In the real world, since paxlovid treatment can significantly reduce the rate of severe COVID-19 and mortality, especially in older patients, it is undoubtedly the first choice of the most cost-effective and effective treatment for mild to moderate patients. Therefore, it is important to screen for effective drugs for COVID-19 patients who progress to severe disease.

The use of baricitinib in patients with severe COVID-19 has been found to result in early stabilization of lung function, reduced need for intensive care support, and reduced re-hospitalization and mortality.^[17]^ Additional studies have shown that baricitinib combined with corticosteroids improves lung function to a greater extent than corticosteroids alone in moderate-to-severe COVID-19 patients and has been shown to reduce mortality among hospitalized COVID-19 patients.^[15,28]^

Other systematic reviews and meta-analyses evaluating baricitinib have produced similar results. A recent meta-analysis reported that baricitinib treatment reduced 28-day mortality in hospitalized patients with COVID-19, with no significant reduction in the proportion of patients requiring MV.^[29]^ Another meta-analysis showed that baricitinib improved the ICU admission rates, mechanical ventilation demand, and oxygenation.^[30]^ However, our results have not been as consistent as those of some clinical studies of baricitinib. Our analysis showed that the length of hospital stay for severe COVID-19 patients treated with baricitinib did not differ from that of standard care. We speculate that this is most likely related to the higher mortality rate in the standard treatment group and non-achievement of the primary outcome of progression to invasive mechanical ventilation, death, or recovery in baricitinib patients by the end of the trial. In terms of the incidence of serious adverse reactions, baricitinib did not show any advantage over the standard treatment group, which we believe is related to the occurrence of a human inflammatory factor storm and various complications in the severe stage of COVID-19.^[22]^ However, we have not seen evidence that baricitinib exacerbates these adverse events or raises safety concerns.

Unlike tocilizumab, baricitinib can be administered orally, is easy to store, and is much less expensive for a shorter period of use. These factors qualify it for priority use in low-income or middle-income countries.^[31]^ The Infectious Diseases Society of America (IDSA) has also issued a modest recommendation for the use of baricitinib, remdesivir, and corticosteroids in patients with severe or critically ill COVID-19 infections. In February 2022, the US National Institutes of Health (NIH) updated its guidelines to recommend the use of baricitinib in patients treated with dexamethasone for COVID-19, who require oxygen uptake and a systemic inflammatory response.

As with similar studies, there were inevitable limitations to our meta-analysis. First, each study differed in inclusion criteria, clinical practice heterogeneity across geographic areas, and measured outcomes. Therefore, there was significant heterogeneity in the statistical analysis. Second, most of the included studies were observational cohort studies, which implies a high selection bias owing to the lack of blinding of participants and personnel interventions. Third, various treatment regimens were used in those studies, which made the dose and frequency of the intervention drugs inconsistent.

However, the strength of our study lies in the inclusion of all the most recent studies, the large sample size of patients, and robust and reliable analytical methods. In conclusion, the results of our meta-analysis strengthen the evidence that baricitinib reduces mortality and advances mechanical ventilation in patients with severe COVID-19, which has important implications for guiding clinical practice.

## Author contributions

**Formal analysis:** Tianqi Gao.

**Investigation:** Wenxin Song.

**Methodology:** Wenxin Song.

**Project administration:** Yilong Feng.

**Software:** Shishen Sun.

**Supervision:** Shishen Sun, Jie Chen.

**Validation:** Liujun Liu, Shaoxiang Xian.

**Visualization:** Liujun Liu, Shaoxiang Xian.

## Supplementary Material








