# Supplementary material for: Efficacy and safety of baricitinib in patients with severe COVID-19: A systematic review and meta-analysis
Source: Medicine (Baltimore). 2023 Dec 1;102(48):e36313. doi: 10.1097/MD.0000000000036313 (PMC10695502; doi:10.1097/MD.0000000000036313)
Supplement: Supplementary file 4 [file medi-102-e36313-s004.docx]

**Table S4. GRADE Evidence Quality Assessment for Rates of Severe Adverse Events (Including Venous Thromboembolism Events, Acute Kidney Failure, and Severe Allergic Reactions).**

| Regimen of experimental group | Regimen of control group | Limitation | Imprecision | Heterogeneity and inconsistency | Indirectness | Publication bias | GRADE |
| --- | --- | --- | --- | --- | --- | --- | --- |
| Baricitinib 4mg/d up to 14 days or until discharge from hospital (whichever occurred first) and standard of care | Placebo for up to 14 days or until discharge from hospital and standard of care | No downgrade | No downgrade | No downgrade | No downgrade | No downgrade | ⨁⨁⨁⨁  High |
| Baricitinib 4 mg/d for 14 days and dexamethasone 6 mg/d i.v. for 10 days and standard of care | Dexamethasone 6 mg/d i.v. for 10 days and standard of care | No downgrade | No downgrade | No downgrade | No downgrade | No downgrade | ⨁⨁⨁⨁  High |
| Received corticosteroids for 3 days and then prednisone, combined with baricitinib for 5–10 days. Baricitinib: 4 mg the first day and then 2mg/d (n =40) or 4/d (n=22). | Received three consecutive days of pulse corticosteroid therapy (corticosteroids pulses) followed by prednisone at a starting dose of 30 mg/d. | Downgrade^1^ | No downgrade | No downgrade | No downgrade | No downgrade | ⨁⨁⨁◯MODERATE |
| Baricitinibs plus tocilizumab plus dexamethasone plus remdesivir plus standard of care | Tocilizumab plus dexamethasone plus remdesivir plus standard of care | Downgrade^1^ | No downgrade | No downgrade | No downgrade | No downgrade | ⨁⨁⨁◯MODERATE |
| Baricitinib 8 mg/d for 14 days plus dexamethasone 0.25 mg/kg/d i.v. plus remdesivir (200 mg loading followed by 100 mg once daily) plus standard of care | Baricitinib 4mg/d for 14 days plus dexamethasone 0.25 mg/kg/d i.v. plus remdesivir (200 mg loading followed by 100 mg once daily) plus standard of care |  |  |  |  |  |  |
| Baricitinib plus other drugs were given in various combinations at the discretion of the attending physician | Drugs were given in various combinations at the discretion of the attending physician | Downgrade^1^ | No downgrade | No downgrade | No downgrade | No downgrade | ⨁⨁⨁◯MODERATE |
| 1. Over 70% of the contribution originates from comparisons with moderate Risk of Bias (RoB).  2. This is due to the fact that less than 30% of the contribution stems from comparisons with low RoB.  3. This is attributed to the point estimate being less than 1.0 while the upper limit exceeds 1.25. | | | | | | | |
